# Self-inflicted ingestion of harmful chemicals in adolescents and adults: risk factors and characteristics

**DOI:** 10.1186/s12889-024-18971-3

**Published:** 2024-06-20

**Authors:** Rajen Govender, Ashley van Niekerk, Tiffany Joy Hector, Wayne van Tonder

**Affiliations:** 1 Institute for Social and Health Sciences, University of South Africa, South East Metropolitan Complex, Lenasia, Johannesburg, 1827 South Africa; 2 Violence, Injury, and Social Asymmetries Research Unit, University of South Africa and South African Medical Research Council, Violence, Injury, and Social Asymmetries Research Unit, University of South Africa Cape Town Campus, 15 Jean Simonis St, Parow, Cape Town, 7530 South Africa

**Keywords:** Ingestion, Self-inflicted, Paraffin, Chemicals, Alcohol

## Abstract

**Background:**

Injury due to ingestion of harmful chemicals has become an area of concern globally. In South Africa, paraffin has been widely implicated in multiple health outcomes, including severe ingestion injuries. A specific category of such injuries is those that are self-inflicted. A significant proportion of self-inflicted ingestion is reported to be intentional, although intentionality for self-infliction may be difficult to determine. Nonetheless, the identification of key explanatory risks and demographic factors of self-inflicted ingestion may contribute towards a better understanding of self-inflicted and harmful chemical ingestion injuries.

**Methods:**

This study used secondary data that had been collected on burn injuries of all causes, including those due to the ingestion of harmful chemicals, from a sample of South Africans from low-income communities close to major metropolitan centres. The current analysis focused on the risks for self-inflicted ingestion injuries and used logistic regression to determine risks for self-inflicted ingestion as differentiated from ingestion due to the actions of another person (other-inflicted ingestion) by sex and age cohort of the victim, and the presence of alcohol, by examining paraffin ingestion versus that of other chemicals.

**Results:**

The overwhelming majority of ingestion injuries (92.1%) were self-inflicted. The current findings indicate that sex (with females almost twice as likely to present with self-inflicted ingestion), age cohort (with those aged 18–29 and 30–44 years old four times more likely affected than older adults), presence of alcohol (twice as likely present than amongst individuals reporting ingestion injuries inflicted by others), and chemicals other than paraffin (three times more likely) are key explanatory factors for an increased risk for self-inflicted ingestion of harmful chemicals.

**Conclusions:**

The study empirically confirms the role of several key risk factors in what remains a relatively unreported and understudied phenomenon, but which appears to align with the demographic and risk profile reported for suicidal injuries through chemical ingestion, i.e. intentional self-inflicted ingestion. The findings may contribute towards improved safety policies on the availability and sale of chemical products and more focussed community interventions for at-risk individuals such as females and young people. It also flags the importance of assessing for alcohol use and alcohol use disorders at hospital admission of self-ingestion injuries.

## Global distribution, demographics and causality of ingestion mortality

The exposure to harmful chemicals is common and may affect people through ingestion, inhalation, skin contact or via the umbilical cord to unborn children [1]. The World Health Organization (WHO) notes that poisoning “from pharmaceuticals, industrial chemicals, pesticides, chemical products and natural toxins is a significant global public health problem” [2, paragraph 1]. In 2012, global unintentional poisoning mortality was estimated at 154 400 (1.1. per 100 000 in 2019) [3, 4]. The self-inflicted ingestion of pesticides is a further concern that in 2012 accounted for 156 200 deaths. Poisoning injury mortality is concentrated in South East Asia, followed by Sub-Saharan Africa and other regions [4]. However, these estimates derive from limited epidemiological data and thus the actual rates may be even higher [4, 5]. In the past three decades there has been an increased research focus on unintentional poisoning and more recently a greater interest in intentional self-poisoning, especially in low- and middle-income countries [6, 7]. The most prevalent agents of lethal poison ingestion vary by region and country. For example, in a systematic review of lethal poisoning in India (of 186 articles from 1999 to 2018), over 16 500 poisoning fatalities were reported over this period. Pesticide mortality was most prominent (94.5% of deaths) with organophosphorus insecticides the most common agent especially since 2001 [8]. In Sri Lanka, from April 2002-December 2019 there were nearly 35 000 hospital admissions of potential or confirmed pesticide poisoning, with a median age of 29 years and with 66% of cases male [9]. The high prevalence of poisoning with pesticide in India reflects its widespread availability and common agricultural and domestic household use [10]. In China, there was a decrease in poisoning mortality between 2009 and 2016, as indicated by data from all 31 provinces [11]. Poisoning by pesticide was most common and involved in 88% of deaths, most of which were suicides among persons older than 15 years, males, and those living in rural areas [12].

In parts of Africa, intentional poisoning mortality, especially attributed to organophosphates, also poses a major public health problem [13]. In an Ethiopian study, the most common reason noted for intentional poisoning was family disputes, with 24.4% of poisoning cases attributed to the ingestion of bleach of which 71.4% were female [14]. In Zambia organophosphates were most commonly ingested, in 38% of cases, with most cases an attempted suicide following issues in a marriage (24.4%), relational problems (19.1%), psychosocial issues (12.2%) and familial problems (10.7%) [15]. In South Africa, poisoning through the ingestion of noxious substances contributed to 3.4% of all injury deaths, i.e. 1877 of all 54 734 injury deaths in 2017 [16, 17], with the majority of poisonings self-inflicted (863 deaths) or where intentionality was undetermined (645 deaths) [17].

Paraffin has been implicated for its involvement in multiple health outcomes, including poisoning but also burn injuries and inhalation problems [18], but is still used by 2.6 million in South Africa [19] despite concerns about its hazardous nature. The public health ramifications of paraffin use are massive and profound, with about 5 000 annual dwelling fires (with about 80% reported the result of paraffin product use) [20] and 100 000 burn injuries (with paraffin a major cause) [21]. Paraffin has furthermore specifically been implicated as an important agent in poisoning incidents, e.g. in unintentional child poisonings, with 40 000 to 60 000 annual cases estimated [22]. Paraffin-related poisoning is sparsely understood in South Africa. The current poisoning research is primarily based on hospital studies in the main urban centres that highlight the prominence of pharmaceuticals especially paracetamol [23] and tricyclic antidepressants [24], and organophosphate poisoning [13].

This study thus builds upon the emerging poison ingestion work in South Africa and describes the demographic and key risk factors for self-inflicted ingestion cases. Due to limitations of data, the study does not distinguish between intentional and unintentional self-inflicted ingestion injuries, and compares all self-inflicted injuries against ingestion injuries attributable to or inflicted by the actions of others. The research explores how demographic factors identified in the literature – sex and age – as well as key attendant factors such as the presence or absence of alcohol and the use of paraffin versus other chemicals may differentiate the risk for self-inflicted from other-inflicted ingestion injuries.

## Methods

### Primary data collection

The primary data for this study was collected by the Paraffin Safety Association of Southern Africa (PASASA) (later renamed Household Energy Safety Association of Southern Africa - HESASA) as part of a larger study on household energy-related risks for mortality and morbidity in South Africa. The dataset for this analysis was derived from a sample of 19 hospitals servicing primarily low-income informal settlements situated nearby major metropolitan areas in eight of the country’s nine provinces. The hospitals are not representative of all hospitals in the country but all of them are located adjacent to and service impoverished communities characterised by informal homesteads. Participants for the study were interviewed at admission in their home language to obtain their informed consent and collect background information on demographics and injury circumstances. Due to low literacy levels amongst the participants, informed consent was obtained verbally. Following admission, participant records were populated by researchers with data obtained from hospital case records, providing detail on the clinical features of the burn injuries and treatment procedures and outcomes. The data was fully anonymised and all participant records were expunged of any form of personal identification information. The study was approved by the Health Research Ethics Committee (HREC) of the South African Medical Research Council.

### Sample

The data for this study were drawn from a larger dataset on paraffin use injuries at the 19 hospitals. The larger study dataset contains 12 079 cases comprising all types of energy-related injuries, in particular external and internal burns, including those due to toxic ingestion, for patients of all ages. Given the focus in this research on ingestion injuries in persons who are adolescents and older, a sub-sample selection was made from the larger dataset using only cases for which ingestion was indicated as the cause of the injury and for which the patient age was reported as 13 years or older. This realised a final analysis sample for this study of 1 718 cases of non-fatal poisonings.

### Analysis

Descriptive statistics were used to illustrate key sample characteristics and binary logistic regression analysis modelling was undertaken to examine for differentiated risk for self-inflicted ingestion across a series of control and key explanatory factors. To account for any potential model estimation problems due to small cell size, the logistic regression analysis was conducted using the bootstrapping method. The bootstrapping method is a procedure whereby the model and parameter estimation is undertaken on multiple different samples drawn from the same base sample. This analytic method returns more robust parameter estimates for each variable. The bootstrapping procedure used in this analysis entailed the drawing and testing of 1000 samples from the base sample. It was conducted by using the bias corrected and accelerated (BCa) intervals rather than the percentile intervals as these provide more robust estimation of the 95% confidence intervals. All Odds Ratio values reported are adjusted for all other variables in the model. The -2 Log Likelihood test was used to assess the significance of both the overall regression model and performance of individual explanatory variables. All analysis was performed using the IBM Statistical Package for Social Sciences (SPSS) version 27, using a *p* ≤ .05 significance level.

### Outcome and explanatory variables

Outcome Variable - the outcome variable was a binary categorical variable differentiating between self-inflicted and other-inflicted ingestion of harmful substances. To sharpen the definition of the other-inflicted ingestion, all instances which were intentional, that is, which would be regarded as assault, were removed from the data. The other-inflicted category thus represents only cases of unintentional infliction of poison ingestion in the victim by other persons. For the category of self-inflicted, the data did not permit a clear resolution of intentionality. Accordingly, the category was not differentiated any further and is used throughout this study to reflect all instances of self-ingestion of harmful substances, whether intentional or unintentional. For the logistic regression modelling the reference category for the outcome variable was set as other-inflicted unintentional ingestion injury.

Explanatory variables - the following explanatory variables were employed:

- Sex: Sex was recorded as either female or male (Reference Category).
- Age Cohort: Age was used as a categorical variable differentiated into four age cohorts as follows: adolescents: 13–17 years, youth or young adults: 18–39 years, mature adults: 30–44 years and older adults: 45 years and older (Reference Category).
- Presence of Alcohol: The presence or absence of alcohol was based on reporting at time of admission by patient and/or persons accompanying patient and/or observation by hospital staff, and was measured categorically as either Present or Not Present (Reference Category). The measure does not include information on prior history of use/abuse or related biomarker testing at admission or during hospitalisation.
- Paraffin and Other Chemicals: This variable was recorded at the time of observation from reporting by the patient or persons accompanying the patient and/or observation by hospital staff and was measured categorically as either Paraffin (Reference Category) or Other Chemical Substances.

## Results

### Descriptives

The total number of participants used for analysis was 1718 (1584 - self-inflicted ingestion, 134 - other-inflicted ingestion). Table 1 demonstrates the variances between those who suffered ingestion of harmful substances through self-inflicted ingestion as compared to those who suffered such injury through other-inflicted ingestion.

**Table 1:** Sample descriptive characteristics

	Self-Inflicted Ingestion 1584 (92.1%)	Other-Inflicted Unintentional Ingestion 134 (7.8%)	Total 1718 (100%)
**Gender**			
Female	81 (6.5%)	1168 (93.5%)	1249 (72.7%)
Male	53 (11.3%)	416 (88.7%)	469 (27.3%)
**Age Cohorts**			
13–17 Years	38 (9.5%)	362 (90.5%)	400 (23.3%)
18–29 Years	54 (6.1%)	833 (93.9%)	887 (51.6%)
30–44 Years	20 (6.2%)	304 (93.8%)	324 (18.8%)
45 Years and Older	22 (20.6%)	85 (79.4%)	107 (6.3%)
**Alcohol**			
Alcohol Present	11 (5.4%)	191 (94.6%)	202 (11.8%)
Alcohol Not Present	123 (8.1%)	1393 (91.9%)	1516 (88.2%)
**Paraffin vs. Other Chemicals**			
Not Paraffin	108 (6.9%)	1451 (93.1%)	1559 (90.8%)
Paraffin	26 (16.4%)	133 (83.6%)	159 (9.2%)

As seen in Table 1, females constituted a much greater proportion of injuries due to ingestion of harmful substances (72.7%) than males (27.3%). Similarly, individuals from the younger age cohorts (13–17 and 18–29 years) comprised the greater proportion of ingestion injuries (74.9%), in contrast to older adults aged 45 years and older (6.3%). The overwhelming majority of ingestion injuries were not characterised by the presence of alcohol (88.2%), with alcohol only occurring in 11.8% of cases. Finally, in terms of the type of chemical substances causing the injuries, the larger proportion was due to the ingestion of chemicals other than paraffin (90.8%) rather than paraffin itself (9.2%).

### Logistic regression analysis

Binary logistic regression analysis was used to determine how the risk for self-inflicted ingestion was differentiated from the risk for other-inflicted ingestion by sex, age cohort, presence of alcohol, and ingestion of paraffin versus other harmful chemicals. The results are presented in Table 2.

**Table 2:** Logistic regression analysis assessing ingestion risk

Self-Inflicted Ingestion	Sig.	Bias Corrected & Accelerated Adjusted Odds Ratios (AOR)	Bias Corrected & Accelerated 95% Confidence Interval for AOR	
			Lower Bound	Upper Bound
Female	0.001	1.961*	1.349	2.895
Male				
13–17 years	0.001	2.730*	1.378	5.129
18–29 years	0.000	4.607*	2.529	8.180
30–44 years	0.000	4.436*	2.207	9.115
45 + years				
Alcohol Yes	0.047	1.918*	1.078	4.558
No Alcohol				
Other Chemicals	0.000	3.057*	1.834	4.697
Paraffin				

The -2 Log Likelihood test returned a statistically significant result for the overall model (χ2 = 56.270, df = 6, *p* = .00), indicating that the model as comprised of all explanatory variables was a good fit to the data. The individual variable -2 Log Likelihood tests further revealed statistically significant results for the explanatory variables in the model as follows: Sex (χ2 = 11.678, *p* = .00), Age cohort (χ2 = 27.634, *p* = .00), Presence of alcohol (χ2 = 4.352, *p* = .03) and Presence of Other Chemicals (other than paraffin) (χ2 = 17.749, *p* = .00).

Inspection of the individual explanatory variables in the bootstrapped model revealed the following:

- Sex – Females were almost twice as likely than males to present at a hospital with self-inflicted ingestion as compared to ingestion inflicted by others (AOR = 1.961, BCa 95% CI: 1.349–2.895). Sex is thus a significant differentiator of the risk of self-inflicted ingestion.
- Age Cohort – When compared to adults aged 45 years or older, young adults aged 18–29 years were most at risk for self-inflicted ingestion, being more than four times more likely to present with self-inflicted injuries compared to the older cohort (AOR = 4.607, BCa 95% CI: 2.529–8.180), followed closely by mature adults aged 30 to 44 years (AOR = 4.436, BCa 95% CI: 2.207–9.115), with adolescents being twice as likely as older adults to suffer such ingestion (OR = 2.730, BCa 95% CI: 1.378–5.129). Overall, all three younger age cohorts are indicated to be at significantly higher risk for self-ingestion, whereas the oldest age cohort is at significantly higher risk for other-inflicted ingestion.
- Alcohol – Individuals for whom the presence of alcohol was recorded for the injury circumstances were twice as likely to suffer self-inflicted ingestion injuries relative to ingestion injuries inflicted by others (AOR = 1.918, BCa 95% CI: 1.078–4.558) Hence alcohol is a significant differentiator of the risk of self-inflicted ingestion from that of other-inflicted ingestion.
- Paraffin vs. other chemicals – finally, self-inflicted injuries were about three times more likely in instances where chemicals other than paraffin were present (AOR = 3.057, BCa 95% CI: 1.834–4.697). This indicates that harmful chemical substances are three times more likely than paraffin to be the cause of injury when the injury is self-inflicted than when it is other-inflicted.

## Discussion

The objectives of this study were to assess all ingestion injuries by three identifying factors, namely (i) the proportion of those that are due to self-ingestion; (ii) the sex and age cohorts most affected; and (iii) the roles of alcohol and other substances. The current findings indicate that sex, age, the presence of alcohol and the ingestion of chemicals other than paraffin were all key explanatory factors with regards to the risk for injuries due to self-inflicted ingestion as compared to ingestion due to the actions of others.

The current findings on the greater risk for self-ingestion of harmful chemicals in females, and thus the likely higher rates of self-harm, align with South African, African and global research and trends on the manner in which high levels of social adversity for women may translate into the higher risk for self-harm behaviour [e.g. 25]. The impact of patriarchal social systems, through social inequality norms, discrimination in essential services such as education and reproductive health, restricted economic opportunities with gender pay gaps [26] and gender-based violence [27], highlight the greater socio-cultural, occupational and personal challenges for women. For example, in Sub-Saharan Africa, nearly half of women (45.60%) reported emotional, sexual and/or physical violence perpetrated by their partners at some point in their life [28]. In South Africa, high rates of gender-based violence, with 266.97 per 100 000 new cases of interpersonal violence for females in 2019, are reported to enforce gender hierarchies [29]. The pervasive challenges of violence faced by girls and women in South Africa [30] and elsewhere [31] are therefore unsurprisingly associated with elevated levels of personal distress, hopelessness helplessness and depression, and consequently, the greater likelihood of self-harm [31].

This study also highlighted age as an important indicator for the greater risk of self-inflicted ingestion, with the highest incidence observed in young adults aged 18 to 29 years, followed by youth aged between 13 and 17, and then mature adults aged between 30 and 44. The result for youth reflects public health concerns for this and equivalent cohorts in other settings. An international review reported that motives for suicide amongst adolescents included internal conflicts and challenges; sociocultural aspects such as socioeconomic difficulty or not being able to access support; relational issues and conflicts; and historic factors such as past trauma [32]. This period in life is characterised, although not necessarily universally, by significant physical, cognitive-emotional, and relational changes [33]. The increased socio-cultural demands that are encountered during this period may be highly stressful and accompanied by feelings of helplessness, burdensomeness, shame and a loss of control, with such feelings commonly manifest in suicide attempts [32, 33], and exacerbated by alcohol and substance use [32]. This study confirmed the role of alcohol in differentiating the risk of self-inflicted ingestion from that of other-inflicted ingestion, doubling the risk for self-inflicted injuries. This result is consistent with other research reporting on the association between alcohol and self-ingestion behaviour with a harmful substance, where e.g. in a Sri Lanka study about pesticide poisoning, there was a higher likelihood of over 5 times of the person with self-inflicted poisoning being alcohol dependent [34]. In South Africa, alcohol availability and usage is widespread with binge drinking common amongst young people [35]. Even low doses of alcohol have been found to negatively affect inhibitory control and increase risk-taking behaviour [36] and maladaptive coping [37].

In the current study, mature adults aged 30 to 44 were found to be more than four times more vulnerable than adults aged 45 years and older for self-inflicted ingestion. This cohort is also considered an intensive life period, with significant life demands and events related to adulthood, with employment or unemployment, marriage and family demands, and lifestyle pressures, all of which can impact wellbeing and ultimately suicidal behaviour [33]. In South Africa, the unemployment rate for the last quarter of 2023 was 32.1% [38] with profound socio-economic challenges for both younger and more mature age cohorts, straining the emotional and social capacities of individuals. The struggle to find employment or the loss of employment and income and the impact of this on South African families has been extremely stressful, with individuals who typically carry the responsibility of family care burdened by increased rates of family instability, alcohol use and dependence, interpersonal conflict and violence, and psychological illness [33]. In South Africa, there are furthermore specific health challenges for this age group; in particular HIV prevalence peaks between 30 and 39 years, with the highest rates reported amongst females, with consequent impacts on social stigma and personal coping [33].

Finally, the study highlighted that the use of chemicals other than paraffin was more likely during instances of self-inflicted poisoning. The use of other chemicals over paraffin could reflect perceptions of the potency or impact of such chemicals (e.g. pesticides or paracetamols), as well as their accessibility [13], arguably facilitated by the rise of a DIY culture around pesticides for example [39], which would promote the availability of highly corrosive and harmful substances despite concerns of the latter’s safety [40]. An international systematic review has reported that restrictions on specific poisons are associated with lower suicide rates, thus highlighting the importance of controlling access to selected poisons [41] but also pharmaceuticals.

## Strengths and limitations

This study highlights the phenomenon of self-inflicted ingestion injury, specifically within South Africa. This study had access to large sample data, which is generally rare in resource limited contexts such as lower to middle income countries where such data collection and distribution is minimal. Nevertheless, the data used in this study reflect collection from the year 2011 at the very latest, and hence may have a very limited usage life beyond the current period due to changing societal and household conditions. Additionally, while the data enabled the differentiation of self-inflicted from other inflicted injury, it did not permit an examination of intentionality within the category of self-inflicted injury. Accordingly, it is not possible to know the proportion of self-inflicted injury with is attributable to volition and that which is due to negligence or carelessness. This constrains the application of the results to the wider research and literature on self-harm ingestion and highlights the need for further exploratory and empirical research into the intentionality of self-ingestion, especially in settings where such incidents appear to be concentrated.

## Conclusions

This study contributes towards the underreported phenomenon of self-ingestion injury. This has been identified as a knowledge gap in the field, and this study thus sheds light on this complex issue by contributing towards a more nuanced understanding of this phenomenon. Achieving a fuller understanding around self-inflicted ingestion may serve to inform future safety policies on the manner of how chemical products are sold, and the ways in which future prevention and psychosocial interventions can target this area of intentional self-inflicted ingestion injuries. It also provides some recommendations for the triage process at hospitals during admissions, whereby self-ingestion can be noted as a marker for potential underlying or co-occurring mental health issues and the need for relevant psychotherapeutic or psychiatric treatment interventions.

## Data Availability

The data was obtained from the Household Energy Safety Association of Southern Africa (HESASA), a public benefit organisation located in Cape Town, South Africa. Access to the HESASA data is available via direct request to the organisation.
